# Fine particle sources and cognitive function in an older Puerto Rican cohort in Greater Boston

**DOI:** 10.1097/EE9.0000000000000022

**Published:** 2018-09-12

**Authors:** Renee Wurth, Marianthi-Anna Kioumourtzoglou, Katherine L. Tucker, John Griffith, Justin Manjourides, Helen Suh

**Affiliations:** a Department of Nutrition, Harvard T.H. Chan School of Public Health, Harvard University, Boston, Massachusetts; b Department of Environmental Health Sciences, Mailman School of Public Health, Columbia University, New York, New York; c Department of Biomedical and Nutritional Sciences, University of Massachusetts, Lowell, Massachusetts; d Department of Civil and Environmental Engineering, Tufts University, Medford, Massachusetts

**Keywords:** Air pollution, Peurto Rican, Minority health, Cognitive health, Particulate matter

## Abstract

**Background:**

Puerto Ricans living in the mainland US have substantially higher rates of impairment to cognitive performance as compared to non-Hispanic Whites, with air pollutant exposures a potential risk factor. We investigated whether exposures to specific air pollution sources were associated with performance across several cognitive domains in a cohort of Puerto Rican older adults.

**Objectives:**

To investigate the association between sources of fine particulate matter (PM_2.5_) and cognitive performance in each of five cognitive domains.

**Methods:**

We obtained demographic, health, and cognitive function data for 1500 elderly participants of the Boston Puerto Rican Health Study. Cognitive function was assessed in each of two waves for five domains: verbal memory, recognition, mental processing, and executive and visuospatial function. To these data, we linked concentrations of PM_2.5_ and its components, black carbon (BC), nickel, sulfur, and silicon, as tracers for PM_2.5_ from traffic, oil combustion, coal combustion, and resuspended dust, respectively. Associations between each PM_2.5_ component and cognitive domain were examined using linear mixed models.

**Results:**

One year moving average exposures to BC were significantly associated with decreased verbal memory (−0.38; 95% confidence interval [CI] = −0.46, −0.30), recognition (−0.35; 95% CI = −0.46, −0.25), mental processing (−1.14; 95% CI = −1.55, −0.74), and executive function (−0.94; 95% CI = −1.31, −0.56). Similar associations were found for nickel. Associations for sulfur, and silicon, and PM_2.5_ were generally null, although sulfur (−0.51; 95% CI = −0.75, −0.28), silicon (−0.25; 95% CI = −0.36, −0.13), and PM_2.5_ (−0.35; 95% CI = −0.57, −0.12) were associated with decreased recognition.

**Conclusion:**

Long-term exposures to BC and nickel, tracers of traffic and oil combustion, respectively, were associated with decreased cognitive function across all domains, except visuospatial function.

## Abstract 2

Supplemental Digital Content is available in the text.

**What this study adds**

While a growing number of studies have examined the impact of particulate matter (PM) on cognition, relatively few studies have examined the role of PM sources and their components on cognition, with even fewer studies examining these impacts on minority communities who may be most vulnerable to these impacts. To address these knowledge gaps, we examined associations of PM sources and cognition across multiple domains in a cohort of Puerto Rican older adults living in metropolitan Boston, Massachusetts. We found that PM components related to traffic and oil combustion were consistently associated with lower cognitive function in this potentially vulnerable population.

## Introduction

Cohort studies show that the average prevalence of mild cognitive impairment in the United States ranges from 19% to 28%, with the common finding of increased impairment with age.^1^ The percentage of older adults with cognitive impairment is even higher among Puerto Ricans, for whom the prevalence of cognitive impairment (49% for adults 60+ years) is approximately three times higher than that for non-Hispanic whites residing in the United States.^2^ Factors contributing to the higher cognitive impairment rates in Puerto Ricans are not well understood but may be related to their greater prevalence of risk factors for cognitive impairment, including type 2 diabetes and cardiovascular disease and their generally poorer health.^3^ Puerto Ricans living in the mainland US, for example, have the highest prevalence of diabetes (26.1%) and the greatest activity limitation, compared to other Hispanic groups.^4^ Additionally, Puerto Ricans living in metropolitan Boston have been shown to have higher rates of obesity (56%) and hypertension (69%) as compared to their non-Hispanic white counterparts.^2^

Even though prevalence of these conditions is higher among Puerto Ricans, in non-Hispanic white populations, it has been shown that these conditions alone do not fully explain the prevalence of cognitive impairment.^3,5,6^ It is, thus, also unlikely to fully explain the high prevalence among Puerto Ricans, suggesting a role for other risk factors such as exposure to airborne fine particulate matter (PM_2.5_). Exposure to fine particles (PM_2.5_; particles with aerodynamic diameter ≤2.5 μm) has been shown to be higher in Hispanics as compared to other populations^7^ and, further, has been linked to adverse cardiovascular outcomes^8,9^ and, more recently, to cognitive impairment.^10–15^ For example, exposures to PM_2.5_ were associated with worsening episodic memory in the Health and Retirement Survey,^16^ decline in global cognition in Nurses’ Health Study participants,^15^ and greater cognitive decline in the Americans Changing Lives Study.^17^

Notably, some components and sources of PM_2.5_ have been shown to be more harmful than others. Exposure to traffic-related pollutants, such as black carbon (BC) or elemental carbon, have been positively associated with cardiovascular-related hospital admissions and mortality,^18–21^ and exposures to vanadium, a tracer of oil combustion, and silicon, a proxy of crustal particulate matter, have been associated, albeit less consistently, with cardiovascular admissions.^18,22^ While not well studied, it is possible that PM_2.5_ components and sources also have differential impacts on cognitive performance, given the documented connection between risk factors for cardiovascular and cognitive disease^3,5,6^ and evidence showing associations between traffic-related exposures and cognitive performance in older Americans.^11,13,15,23,24^

To investigate the association between PM_2.5_ and cognitive performance, we examined the association between PM_2.5_ and its components and performance on tests of cognitive function, using demographic, health, and cognitive function data from a cohort of Puerto Rican older adults participating in the Boston Puerto Rican Health Study (BPRHS). We specifically focused on examining the impact of PM_2.5_ components that have been shown to be tracers for traffic, oil combustion, coal combustion regional pollution, and crustal sources because these sources are known to be the key sources of PM_2.5_ in Boston and elsewhere. As these components (and other pollutants that originate from their associated sources) may affect cognition through different pathways, we assessed their effects on five specific cognitive domains: verbal memory, recognition, mental processing speed, executive function, and visuospatial function.

## Methods

BPRHS is an ongoing, longitudinal study designed to examine the role of psychosocial stress on presence and development of allostatic load and health outcomes in Puerto Ricans. In this study, numerous self-reported and biological measures of physiologic and cognitive health were collected in each of two data collection waves (2004–2008 and 2008–2012) for 1,500 Puerto Rican older adults (aged 45–75 years) living within metropolitan Boston, Massachusetts.^2^ There was a median of 2 year difference between waves, with 1258 participants contributing to both waves. All procedures involving human subjects were approved by the Institutional Review Board at Tufts Medical Center and Northeastern University. Written informed consent was obtained from all subjects.

### Participant characteristics

Participants provided information on age, education level, and employment history via interviewer-administered questionnaires.^2^ Body mass index was calculated using weight (kg) divided by height (m) squared. Systolic and diastolic blood pressures were measured in duplicate, at three time points during the interview, and averaged. Income to poverty ratio was calculated as the total household income divided by the poverty threshold for that sized family (using poverty guidelines 2004–2007).

### Cognitive measures

Cognitive performance was assessed for each participant in each of the two waves through a comprehensive neuropsychological examination comprising five tests: the California Verbal List Learning (List Learning), Stroop, Letter Fluency, clock drawing, and figure copying tests. Tests were selected based on their documented validity in Spanish-speaking populations and in neuropsychological studies.^2,25–28^ The five tests are well validated and were used to assess performance in five cognitive domains, including verbal memory, mental processing speed, executive function, and visuospatial function.^2^

A trained interviewer administered each test in a set sequence, as ordered below, during the home visit for each of the data collection waves. Tests were administered in English or Spanish, based on the preferred language of the participant. The majority of participants completed the cognitive tests (1127 or 90% participants completing all five tests in both waves). While still high, the mental processing test had the lowest completion rate, with a 92% and 90% completion rate in Wave 1 and 2, respectively. The characteristics of participants completing the tests generally did not differ from other participants, except for educational attainment and diabetes history, for which individuals completing the mental processing test had higher educational attainment and reported history of diabetes. In total, complete cognitive function data were available for 1225 (of the 1497) participants in Wave 1 and for 1233 (out of 1258) participants in Wave 2.

#### Verbal memory.

The California Verbal List Learning test is one of the five most widely used neuropsychological tests^30^ given its ability to test short-term, long-term, and other aspects of verbal memory and its well-documented reliability^31^ (r = 0.62) and validity.^26,32,33^ In this test, two lists of 16 words are presented to participants, List A and List B. List A is immediately recalled for five consecutive trials to assess short-term retrieval. Short-term retrieval is scored by averaging the scores of these five recall trials from List A (maximum score of 16). Long-term memory is assessed through a process of interference, with List B being presented and followed by free and cued recall of List A and then finally accessing List B again with free and cued recall. The long-term List Learning test is calculated using the average scores for the two delayed recalls and two delayed recall with cues trails of List A, for a maximum score of 16.^34^ For this study, as the main measure of memory, we averaged the scores from the short- and long-term portions of the test to obtain an overall memory score (total list learning), given the high correlation between the short- and long-term memory scores (r = 0.72), as has also been observed in other studies.^16^

#### Recognition.

Recall discriminability, or recognition, was assessed by presenting the original List A as well as 28 distractors to the participants and asking them to recognize the words from the original list. This test component assesses the ability to detect true positives from false positives in the recall list.^35^ Recognition was scored as the number of correct responses (from 1 to 16 total points).

#### Mental processing speed.

The Stroop test, named after its test inventor Dr. J. Ridley Stroop, measures mental processing speed by asking participants to read a list of colors. The Stroop has been used in over 400 studies to test processing speed and was shown to have high validity and reliability when 18 of the most salient studies were reviewed.^27,36,37^ The test involves three trials: word naming, color naming, and color–word naming score.^27^ Results from the Stroop test were scored using the color–word naming score (commonly referred to as Stroop III), which measures the interference of conflicting word stimuli on naming colors, given its higher test–retest reliability when compared with frequently used ipsative (i.e., forced choice) scorings.^38^ The color–word naming score is the count of number of words correct during the 45-second period.^27^

#### Executive functioning.

The letter fluency test evaluates executive function as well as language and verbal fluency. This test is commonly included in neuropsychological assessments, given its high validity in supporting the diagnosis of a wide range of diseases, such as types of dementia and Alzheimer’s disease.^39,40^ It is a phonemic category test, giving participants 1 minute to list words that start with a given letter. This test is performed using three different letters, with the score based on the total number of words identified in these three trials.

#### Visuospatial function.

Visuospatial function was assessed using the clock drawing and figure copying tests that jointly evaluate visual and spatial memory, processing, and reason. Clock drawing scores participants on their ability to draw a clock with one point given for including all 12 numbers, in correct position, and with hands in position on clock. Due to its high degree of sensitivity and specificity, clock drawing has been shown to detect executive functioning changes that cannot be detected by other tests, such as the Mini Mental Status Exam, making it complimentary to the letter fluency test.^28,41^ The figure copying test asks participants to replicate nine figures, which are scored to provide a total of 12 possible points. It is unique in its well-studied sensitivity to Alzheimer’s disease.^42^ The average of the scores of the clock drawing and figure copying tests was used as the measure of visuospatial function.

### Air pollution exposure assessment

#### Measurement.

Ambient concentrations of PM_2.5_ and its components BC, nickel, sulfur, and silicon were measured at the US Environmental Protection Agency PM Center stationary ambient monitoring supersite, located in downtown Boston, Massachusetts, on the roof of Countway Library at the Harvard Medical School. BC concentrations were measured every 5 minutes using an Aethalometer (model AE-14 by Magee Scientific, Berkeley, CA). The 24-hour integrated PM_2.5_ samples were collected using a Sequential Sampler (Partisol Model 2300 by Rupprecht and Patashnick, Albany, NY) at a flow rate of 16.7 LPM. PM_2.5_ samples were analyzed for mass using gravimetric analysis and for elemental concentrations using X-Ray Fluorescence.

#### Exposure measures.

Major sources of PM_2.5_ in metropolitan Boston were identified using results from Kioumourtzoglou et al.^43^ who apportioned 24-hour averaged PM_2.5_ concentrations into factors that was subsequently corresponded to major source types. From this analysis, we identified tracers for the four sources that showed the largest contribution to PM_2.5_ in Boston by selecting the components that loaded most heavily on these sources. Based on this identification, we included BC, nickel, sulfur, and silicon as tracers for traffic, regional or oil combustion, coal combustion, and crustal PM_2.5_ sources, respectively.

For each participant, we assessed exposures by averaging daily concentrations of BC, nickel, sulfur, silicon, and PM_2.5_ to calculate 1-year and 2-year average exposures ending at the date of each of his/her cognitive exams, with these measures serving as our primary and secondary exposure measures, respectively. Exposure windows were selected based on findings from previous studies of pollutant exposure and cognition.^11,44,45^ One-year and 2-year average exposures were considered valid provided that 75% of the daily values were available, which was the case for all pollutants. Interquartile ranges for each pollutant were calculated for each exposure window for each of the data collection waves. Note that given the design of this study and that exposure assignment was based on concentrations measured at a single monitor, the estimated effects reflect temporal and not spatial contrasts.

### Statistical approach

Given the longitudinal study design, linear mixed models with random intercepts for participant, to account for within participant clustering, were used to assess the association of PM_2.5_ and each PM_2.5_ tracer and each cognitive domain in separate models. Since cognitive performance norms for the cognitive tests have not been established within a population comparable to our Puerto Rican cohort,^46,47^ we treated cognitive function for each test as a continuous outcome, given the lack of meaningful indicators or known clinically relevant cutoffs for cognitive impairment for this cohort. Although missingness in our study was low, below 10% missing for each variable, missing data for all variables used in models were imputed using the Expectation-Maximization algorithm for maximum likelihood parameter estimations, and 95% confidence intervals were calculated using the bootstrap method.^48,49^

Models were adjusted for age, sex, season, physical activity, education, and income-to-poverty ratio.^50^ Adjustment for temperature in models^51^ had no effect on parameter estimates or model fit and, thus, was not included in the final model. To further investigate possible confounding by total PM_2.5_, we performed analysis that adjusted models of BC, nickel, sulfur, and silicon for PM_2.5_. Additionally, we fit two-pollutant models that included BC and either nickel, sulfur, or silicon to examine potential confounding of the BC–cognitive association by nickel, sulfur, or silicon. To compare the magnitude of effects across cognitive domains, we fit models using z-scored results for the examined cognitive domains (eTable 3; http://links.lww.com/EE/A14). A sensitivity analysis looking at the change in waves was also performed to compare to the findings from other models (eTable 4; http://links.lww.com/EE/A14). All statistical analyses were conducted using SAS version 9.4 software (SAS Institute, Inc., Cary, NC). Statistical significance was assessed based on a *P* value of 0.05, unless otherwise noted.

## Results

We analyzed data for all 1497 BPRHS participants who participated in cognitive testing, including 1497 and 1255 participants in Waves 1 and 2, respectively, with 1255 individuals participating in both waves (eTable 1; http://links.lww.com/EE/A14). Participants were similar across tertiles of BC exposure for Wave 1 (Table 1), with additional comparisons across tertiles for other pollutants available in supplemental tables (eTable 5–8; http://links.lww.com/EE/A14). More than 70% of participants were female, with a mean (SD) age of 56.3 (7.7), 58.6 (6.9), and 56.2 (8.0) years in lowest, middle, and highest tertile of BC exposure, respectively. We observed the largest difference between tertiles of BC exposure, with 31.6% of those with the lowest level of exposure having had less than 8th grade education, compared to 39.9% in the middle tertile of exposure (Table 1).

**Table 1:**
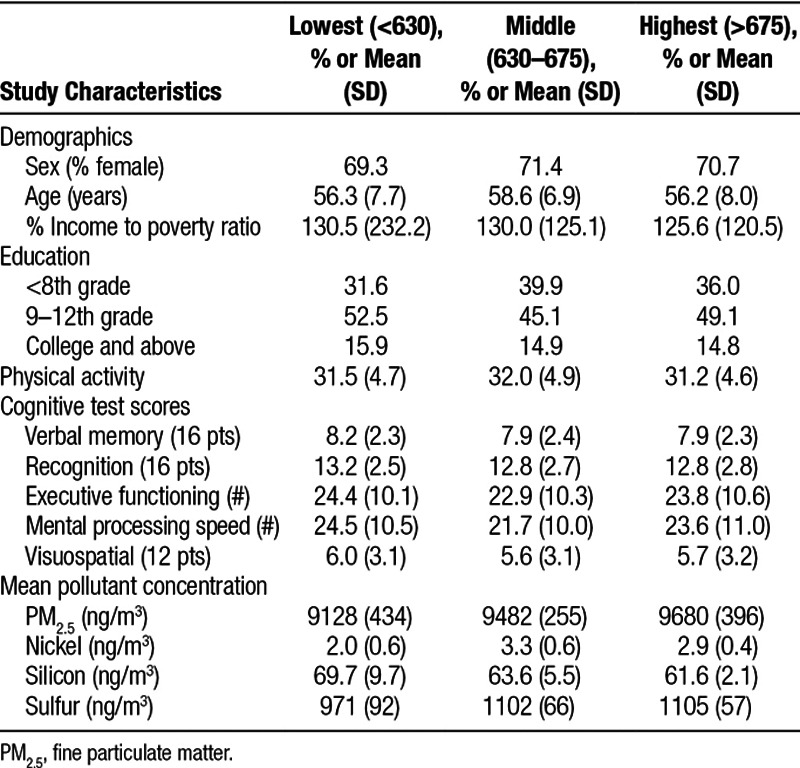
Summary of participant characteristics and pollutant concentrations by tertile of 1 year average of black carbon (ng/m^3^)

Intraclass correlation coefficients for between-wave cognitive test scores ranged between 0.33 (for recall discriminability) and 0.71 (for visuospatial function). The 1-year mean (SD) concentration for BC equaled 665 (85) ng/m^3^ for Wave 1 and 610 (96) ng/m^3^ for Wave 2 (eTable 1; http://links.lww.com/EE/A14). One-year average concentrations (SD) of nickel, sulfur, silicon, and PM_2.5_ concentrations in Wave 1 were 2.8 (0.8), 1077 (90), 64.4 (6.9), and 9781 (896) ng/m^3^, respectively, with lower averages for all pollutants in Wave 2. One-year moving average BC concentrations were significantly and positively correlated with nickel, sulfur, and PM_2.5_ and were negatively correlated with silicon, with Pearson correlation coefficients equal to 0.47, 0.51, 0.37, and −0.34, respectively. In comparison, PM_2.5_ was significantly correlated with BC (r = 0.37), nickel (r = 0.72), and sulfur (r = 0.95), but not silicon (r = 0.19) (eTable 2; http://links.lww.com/EE/A14).

The association between 1-year average exposures to PM_2.5_ and its components BC, nickel, sulfur, and silicon and the five cognitive domains are presented in Table 2. BC, a tracer of traffic, was consistently associated with decreased cognitive function, with significant negative associations found for all domains except for visuospatial function (Table 2). Specifically, in fully adjusted models, an interquartile range (IQR; 53.0 ng/m^3^) increase in 1-year average BC was associated with statistically significant decreases in verbal memory (−0.38; 95% confidence interval [CI] = −0.46, −0.30), recognition (−0.35; 95% CI = −0.46, −0.25), mental processing speed (−1.14; 95% CI = −1.55, −0.74), and executive functioning (−0.94; 95% CI = −1.31, −0.56). While we also observed a negative association between BC exposures and visuospatial function (−0.03; 95% CI = −0.14, 0.07), this decrease was not statistically significant. Of the cognitive domains, BC exposures had the largest impact on verbal memory, with effect estimates twice that of the other tests when models were run using z-scored cognitive test results as the outcome measure (eTable 3; http://links.lww.com/EE/A14). BC-associated cognitive decrements were also found for an IQR increase in 2-year average exposures (eFigure 1; http://links.lww.com/EE/A14). The magnitude of the effect estimates for BC with all cognitive domains increased when adjusting for PM_2.5_ (Table 3). Similarly, the magnitude of the effect estimate of BC with verbal memory, mental processing, and visuospatial function increased in two-pollutant models adjusting for nickel and sulfur (Table 3). For recognition, the effect estimate for BC was attenuated but remained negative and significant in the two-pollutant models.

**Table 2:**
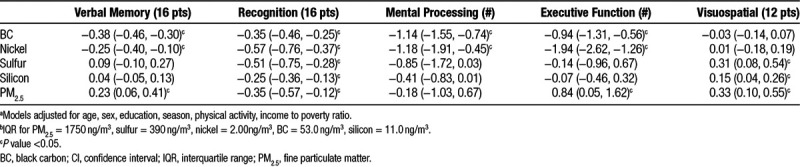
Difference in cognitive score,^a^ per 1-year IQR increase^b^ in source tracer pollutants (β (95% CI))

**Table 3:**
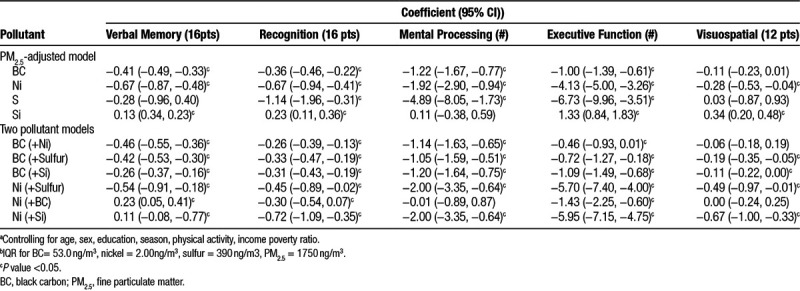
Difference in cognitive score^a^ per IQR increase^b^ in 1-year average BC in PM_2.5_ adjusted and two pollutant models (β 95% CI))

Similar patterns were found in the associations of exposures to nickel and cognitive function. One-year average nickel was significantly associated with decreased verbal memory (−0.25; 95% CI = −0.40, −0.10), recognition (−0.57; 95% CI = −0.76, −0.37), mental processing speed (−1.18; 95% CI = −1.91, −0.45), and executive functioning (−1.94; 95% CI = −2.62, −1.26). As with BC, the magnitude of the association of nickel was greatest for verbal memory, as evidenced by its highest effect estimates when z-scored cognitive test results were used as the outcome variable (eTable 3; http://links.lww.com/EE/A14). The magnitude of the associations were similar when 2-year as compared to 1-year moving averages were used as the exposure measure (eFigure 1; http://links.lww.com/EE/A14). The magnitude of the effect estimates for nickel increased for all cognitive domains in models adjusting for PM_2.5_, were attenuated when adjusting for BC, and increased when adjusting for sulfur exposures (Table 3). In sensitivity analyses exploring the association between changes in particle component concentrations between waves and change in cognitive function, we observed consistent associations for both nickel and BC as in the main analysis, that is, increases in the concentrations of these components were associated with decreases in cognitive function. The results for the other tracer pollutants, however, were less consistent (eTable 4; http://links.lww.com/EE/A14).

Associations for 1-year average exposures to sulfur, a tracer for coal combustion and regional pollution, and silicon, a tracer for crustal PM_2.5_, were inconsistent across the examined cognitive domains. Sulfur was significantly associated with decreased recognition and increased visuospatial function, while silicon was significantly associated with decreased recognition. For other examined cognitive domains, associations were null for both sulfur and silicon.

As was the case for the examined PM_2.5_ components, an IQR increase in 1-year average PM_2.5_ exposure was significantly associated with decreased recognition (−0.35; 95% CI = −0.57, −0.12); however, it was also associated with increased verbal memory (0.23; 95% CI = 0.06, 0.41), executive function (0.84; 95% CI = 0.05, 1.62), and visuospatial function (0.31; 95% CI = 0.16, 0.54). Associations were comparable for 2-year average exposures (eFigure 1; http://links.lww.com/EE/A14).

## Discussion

In our cohort of elderly Puerto Ricans living within metropolitan Boston, exposures to BC and, for the first time, nickel were found to be consistently and significantly associated with decreased verbal memory, recognition, mental processing speed, and executive function but not visuospatial function. Associations of BC and nickel and the examined cognitive domains were consistent across examined exposure windows and were robust to adjustment for PM_2.5_ and for BC, to each other. In contrast, associations of sulfur, silicon, and PM_2.5_ exposures with cognitive function were inconsistent across the cognitive domains and also differed from those observed for BC and nickel, although like BC and nickel, they were each significantly associated with decreased recognition.

Our findings showing significant impacts of BC, and through this traffic-related PM_2.5_, on cognition are consistent with those from previous studies. In other studies conducted in metropolitan Boston, for example, 1-year average exposures to BC were associated with global cognitive decline in elderly men.^11^ Correspondingly, other measures of traffic-related air pollutant exposures have been associated with cognitive impairment, with distance to road negatively associated with global cognitive function in elderly cohorts from Germany and the United States,^13,23^ and NO_2_, with cognitive impairment in elderly cohorts living in Taiwan and Sweden.^52,53^ Significant harmful impacts of air pollution from traffic on working memory and inattentiveness have also been shown for children living in Barcelona, Spain.^54^

Notably, Basagana et al.^54^ did not find significant associations with cognitive function for oil combustion or secondary pollution sources. The null finding for oil combustion differs from our significant findings for nickel, a tracer of oil combustion. Several factors may contribute to this discrepancy, including our study’s focus on older adults as compared to children, our use of nickel concentrations rather than source factors as the exposure measure, and our different measures of cognitive function. Nevertheless, our significant findings for nickel are supported by studies linking nickel exposure to a variety of adverse cardiovascular outcomes in older populations, including those related to increased mortality,^55–57^ hospital admissions,^18,21,22^ inflammation, and atherosclerosis.^58,59^ As nickel, as well as PM_2.5_ and BC, is thought to impact cardiovascular and cognitive health through common biological pathways, such as inflammation, these findings linking nickel exposures to adverse cardiovascular health outcomes provide support for our results showing significant associations between nickel and decreased cognitive function.

In addition to its impacts on inflammation and other pathways, BC and nickel may also impact cognitive function more directly. In animal models, ultra-fine particles and nickel, which like BC, originate from combustion-related sources, have been shown to enter the brain via the olfactory bulb, where they may disrupt the blood–brain barrier, upregulate inflammatory genes and cytokines, and damage the olfactory bulb regions of the prefrontal cortex.^60–62^ Damage near the olfactory bulb regions is consistent with (1) our observed impacts of BC and nickel on verbal memory, recognition, mental processing speed, and executive function, domains which relate to the frontal and prefrontal lobes which sit adjacent to the olfactory bulb,^39,63^ and (2) our null results for visuospatial function, which correlates to the left and right parietal cortices, located further from the olfactory bulb.^64^

While we found consistently significant impacts for BC and nickel on cognitive function, our findings for the other examined pollutants—sulfur, silicon, and PM_2.5_—were largely inconsistent, although notably all pollutants were significantly associated with decreased recognition. Our inconsistent findings for PM_2.5_ are in keeping with findings from several studies of older adults, which show differential impacts of PM_2.5_ depending on the cognitive domain or measure. While several studies of mostly white, higher socio-economic status (SES) cohorts have reported significant associations between PM_2.5_ exposures and decreased general cognitive function,^15–17^ associations are less consistent with specific cognitive domains, such as verbal learning, executive function, memory, and visuospatial function. For example, Gatto et al.^25^ showed PM_2.5_ exposures to be associated with decreased verbal learning, while Schikowski et al.^65^ reported associations of PM_2.5_ exposures and visuospatial ability, but not episodic or semantic memory, executive function, or general cognition. Correspondingly, Tonne et al.^66^ found adverse associations of PM_2.5_ and PM_10_ with reasoning but not with memory or verbal fluency in cross-sectional analyses, and with memory but not reasoning or verbal fluency in longitudinal analyses. Together, these findings suggest that the impacts of PM_2.5_ may differ by cognitive domain, possibly the result of different biological pathways through which different PM_2.5_ components and their sources affect the brain.^60,67^ However, we also observed positive associations between PM_2.5_ and cognitive domains, which we are unable to explain.

Our findings are limited by several factors. First, we assessed air pollutant exposures using measurements made at a stationary ambient monitoring (SAM) site, which has been shown to result in measurement error and lower statistical power.^22^ The magnitude of this measurement error, particularly for the regional pollutants sulfur and PM_2.5_, is likely low, as more than 80% of our participants lived less than 10 km from the SAM site.^68^ This theory is supported by results from Power et al.^11^ that showed spatial heterogeneity of BC concentration near our SAM site to be low, which would also bias toward the null.^43^ Second, cognitive function was measured in only two waves that were conducted relatively close in time, which together with our exposure measures from a single SAM site did not provide sufficient power to test the association between air pollution exposures and cognitive decline among our participants. In sensitivity analyses, however, we showed that changes in cognitive function between waves were significantly associated with Ni and BC, further increasing our confidence in the role of these components and related sources on cognition. Third, our findings are limited by the potential for residual confounding, given that we were not able to control completely and perfectly for socioeconomic status. Our findings may also be limited by statistical issues related to multiple comparisons, given the number of exposures and outcomes investigated in this study. Even with these limitations, the consistency of our findings in both the main and two-pollutant models, for different exposure windows, and across multiple cognitive domains support the validity of our findings.

These limitations are outweighed by our study’s substantial strengths. To our knowledge, this is the first study to investigate the impact of key PM_2.5_ sources on the function of multiple cognitive domains in Puerto Rican adults living in metropolitan Boston, an understudied group who may be particularly susceptible to air pollution’s harmful effects due to their low socioeconomic status and high rates of disease, both of which have been shown to modify the association of air pollution and cognition. In so doing, we showed BC and nickel, tracers of traffic and oil combustion, respectively, to have consistent and significant association with cognitive impairment across nearly all examined cognitive domains. Our findings demonstrate the importance of studying minority and other high-risk populations and identifying modifiable risk factors such as air pollution to lower their high burden of cognitive disease.

## Conflicts of interest statement

The authors declare that they have no conflicts of interest with regard to the content of this report.

## Source of funding

Air pollution concentrations were supported by the US Environmental Protection Agency (EPA) grant RD-83587201. The Boston Puerto Rican Health Study (BPRHS) was supported by the National Institutes of Health through NIH P01 AG023394 and P50 HL105185. Additional support for Drs. Suh and Manjourides was provided by NIEHS grant 1R01ES022657-01A1 and for Dr. Kioumourtzoglou by NIH T32 ES007069.

Description of data: Data can be requested through contacting representatives for the BPRHS at Esther_Carver@uml.edu. Code for reproduction can be obtained through contacting the corresponding author.

Data collection instruments: Boston Puerto Rican Health Study questionnaires are available online at https://www.uml.edu/Research/UML-CPH/Research/projects/bprhs/default.aspx

## Supplementary Material
