# Supplementary material for: Fine particle sources and cognitive function in an older Puerto Rican cohort in Greater Boston
Source: Environ Epidemiol. 2018 Sep 12;2(3):e022. doi: 10.1097/EE9.0000000000000022 (PMC6261496; doi:10.1097/EE9.0000000000000022)
Supplement: Supplementary file 1 [file ee9-2-e022-s001.pdf]

eTable 1. Summary of Participant Characteristics and Pollutant Concentrations by Wave

**Study Characteristics**	**Wave 1 (2004-08)** % or mean (SD)	**Wave 2 (2008-2012)** % or mean (SD)
Demographics		
Sex (% female)	70.4	71.9
Age (years)	57.1 (7.6)	59.3 (7.7)
Income Poverty Ratio		
Education		
<8th grade	35.9	36.5
9-12th grade	48.8	48.9
College and above	15.2	14.6
Behavior		
Physical activity[1]	31.6 (4.7)	31.8 (4.7)
Cognitive Test Scores		
Verbal memory (16 pts)	8.0 (2.3)	8.2 (2.3)
Recognition (16 pts)	12.9 (2.7)	13.4 (2.4)
Executive functioning (#)	23.4 (10.3)	24.4 (11.3)
Mental processing speed (#)	22.7 (10.4)	22.4 (10.5)
Visuospatial (12 pts)	5.7 (3.1)	5.5 (3.2)
Mean Pollutant Concentration		
$PM_{2.5}$ (ng/m^3)	9781 (896)	7742 (654)
Black carbon (ng/m^3)	665 (85)	610 (96)
Nickel (ng/m^3)	2.8 (0.8)	1.0 (0.3)
Silicon (ng/m^3)	64.4 (6.9)	62.6 (15.9)
Sulfur (ng/m^3)	1077 (90)	707 (16)

eFigure 1. Difference in Cognitive Score[1] Per IQR increase[2] in Source Tracers and $PM_{2.5}$ (β(95% CI)) for 1- and 2-Year Moving Averages[3]

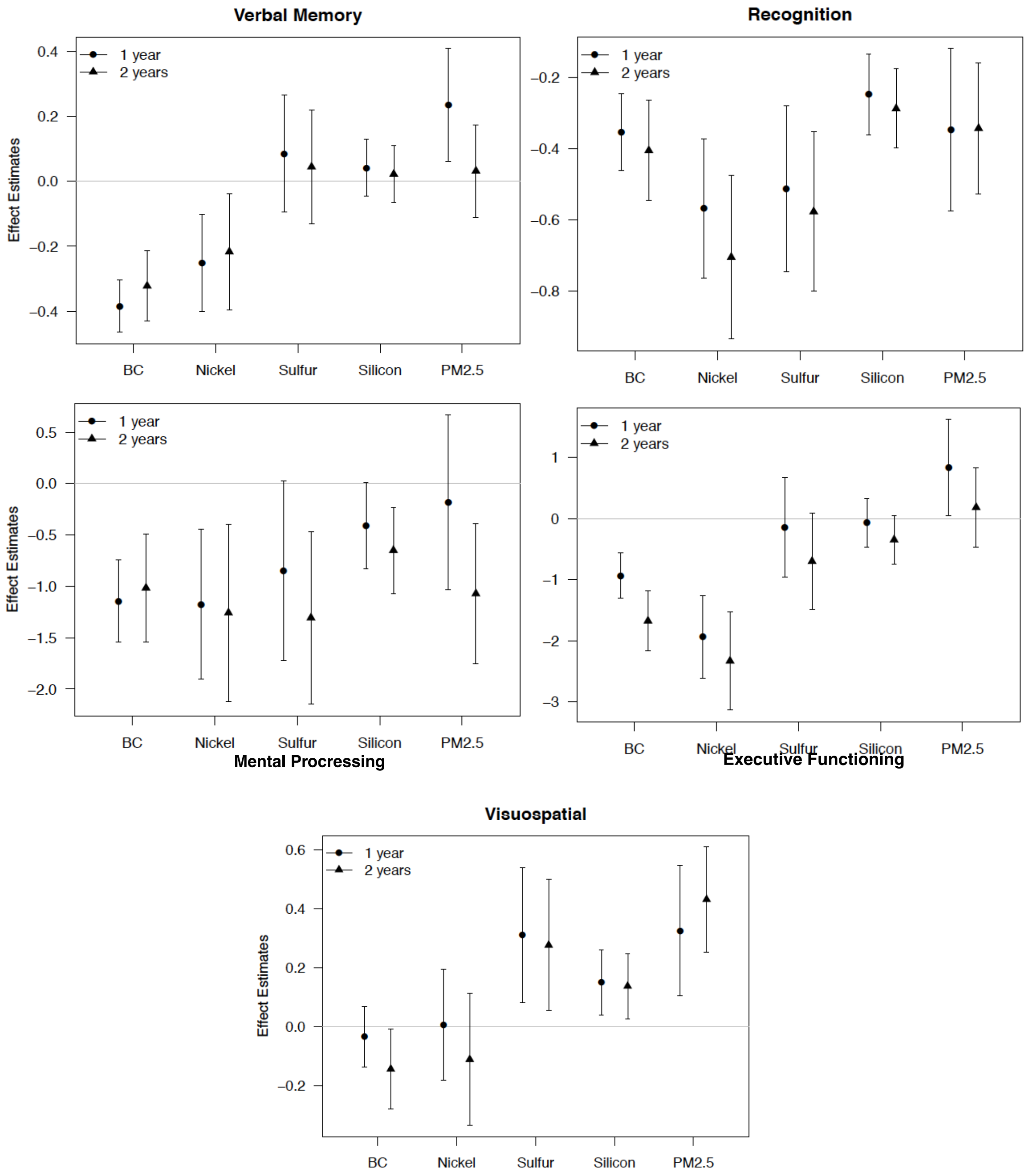

[1]Controlling for: age, sex, education, season, physical activity, income poverty ratio
[2]IQR for BC= 53.0 ng/m^3, nickel=2.00ng/m^3, sulfur =390 ng/m3, $PM2_{.5}$=1750 ng/m^3
[3]Y-axes are scaled according to cognitive test

eTable 2. Pearson Correlation Coefficients Comparing Annual Average Pollutant Concentrations[1]

	BC	Nickel	Sulfur	Silicon
$PM_{2.5}$	0.37*	0.72*	0.95*	0.19*
BC	1.00	0.87*	0.47*	-0.34*
Nickel	--	1.00	0.87*	0.003
Sulfur	--	--	1.00	-0.34*
Silicon	--	--	--	1.00

[1]2003-2012; *p-value<0.05

eTable 3. Difference in Cognitive Z-score[1], Per 1 year IQR increase[2] in Source Tracer Pollutants (β(SE))

	**Verbal Memory** (16 pts)	**Recognition** (16 pts)	**Mental Processing** (#)	**Executive Function** (#)	**Visuospatial** (12 pts)
BC	-0.38 (0.04)**	-0.14 (0.02)**	-0.11(0.02)**	-0.09 (0.02)**	-0.02 (0.02)
Nickel	-0.25 (0.08)**	-0.22 (0.04)**	-0.11 (0.02)**	-0.18 (0.03)**	-0.02 (0.01)
Sulfur	0.08 (0.10)	-0.17 (0.05)**	-0.08 (0.04)	-0.01 (0.04)	0.10 (0.03)**
Silicon	0.04 (0.05)	-0.09 (0.02)**	-0.04 (0.02)	-0.10 (0.02)	0.05 (0.02)*
$PM_{2.5}$	0.23 (0.08)**	-0.13 (0.04)**	-0.02 (0.04)	0.08 (0.04)**	0.82(0.04)**

[1]Controlling for: age, sex, education, season, physical activity, income to poverty ratio
[2] IQR for $PM_{2.5}$ = 1750 ng/m^3, sulfur =390 ng/m^3, nickel =2.00ng/m^3, BC = 53.0 ng/m^3, silicon = 11.0 ng/m^3
p value **<0.05 *<0.10

eTable 4. Change in Cognitive Score, Per IQR[2] increase in Difference between Waves of Source Tracer Pollutants (β(SE))

	**Verbal Memory** (16 pts)	**Recognition** (16 pts)	**Mental Processing** (#)	**Executive Function** (#)	**Visuospatial** (12 pts)
BC	-3.82 (0.50)**	-0.37 (0.18)*	-2.01 (0.49)**	-1.48 (0.40)**	-0.04 (0.18)
Nickel	-1.60 (0.49)**	-0.18 (0.14)	-1.06 (0.41)**	-1.92 (0.39)**	-0.14 (0.12)
Sulfur	2.70 (0.34)**	0.10 (0.08)	-0.16 (0.34)	1.44 (0.24)**	0.43 (0.06)**
Silicon	3.14 (0.46)**	0.26 (0.13)*	-0.89 (0.46)*	3.11 (0.42)**	0.97 (0.11)**
$PM_{2.5}$	3.38 (0.40)**	0.28 (0.11)*	0.27 (0.40)	1.82 (0.61)**	0.47 (0.12)**

[1]Controlling for: age, sex, education, season, physical activity, income to poverty ratio
[2] IQR for $PM_{2.5}$ = 866 ng/m^3, sulfur =113 ng/m^3, nickel =0.71/m^3, BC = 85.6 ng/m^3, silicon = 21.1 ng/m^3
p value **<0.05 *<0.10

eTable 5. Summary of Participant Characteristics and Pollutant Concentrations by Tertile of 1 yr Average of $PM_{2.5}$ (ng/m^3)

Study Characteristics	Lowest (<9300) % or mean (SD)	Middle (9300-9555) % or mean (SD)	Highest (>9555) % or mean (SD)
Demographics			
Sex (% female)	69.3	71.2	70.7
Age (years)	56.1 (7.8)	58.0 (7.2)	57.2 (7.6)
% Income to Poverty Ratio	125.2 (117.0)	128.9 (239.1)	131.0 (128.8)
Education			
<8th grade	32.1	38.8	36.6
9-12th grade	53.0	46.6	47.6
College and above	14.9	14.6	15.8
Physical activity[1]	31.6 (4.7)	31.6 (4.7)	31.5 (4.8)
Cognitive Test Scores			
Verbal memory (16 pts)	8.1 (2.4)	8.2 (2.3)	7.8 (2.3)
Recognition (16 pts)	13.0 (2.6)	13.2 (2.6)	12.7 (2.8)
Executive functioning (#)	23.6(10.3)	23.8 (10.4)	23.0 (10.5)
Mental processing speed (#)	23.7 (10.5)	23.2 (10.7)	22.9 (10.5)
Visuospatial (12 pts)	6.0 (3.1)	5.5 (3.1)	5.9 (3.2)

eTable 6. Summary of Participant Characteristics and Pollutant Concentrations by Tertile of 1 yr Average of Nickel (ng/m^3)

Study Characteristics	Lowest (<2.0)	Middle (2.0-3.0)	Highest (>3.0)

	% or mean (SD)	% or mean (SD)	% or mean (SD)
Demographics			
Sex (% female)	67.9	69.4	72.4
Age (years)	55.6 (7.8)	56.6 (8.1)	58.2 (7.1)
% Income to Poverty Ratio	129.7 (239.7)	117.4 (100.2)	133.0 (133.8)
Education			
<8th grade	30.3	35.3	39.6
9-12th grade	54.9	52.5	43.7
College and above	14.8	12.2	16.8
Physical activity[1]	31.6 (4.9)	31.1 (4.4)	31.7 (4.8)
Cognitive Test Scores			
Verbal memory (16 pts)	8.1 (2.4)	7.9 (2.4)	8.0 (2.3)
Recognition (16 pts)	13.1 (2.6)	12.7 (2.9)	12.9 (2.6)
Executive functioning (#)	24.3 (10.1)	22.9 (10.3)	23.8 (10.6)
Mental processing speed (#)	24.5 (10.6)	22.8 (10.4)	22.6 (10.6)
Visuospatial (12 pts)	6.1 (3.1)	5.5 (3.2)	5.7 (3.1)

eTable 7. Summary of Participant Characteristics and Pollutant Concentrations by Tertile of 1 yr Average of Silicon (ng/m^3)

**Study Characteristics**	**Lowest (<61.2)** % or mean (SD)	**Middle (61.2-63.9)** % or mean (SD)	**Highest (>63.9)** % or mean (SD)

Demographics			
Sex (% female)	68.3	74.3	69.2
Age (years)	57.4 (7.3)	57.8 (7.8)	56.3 (7.6)
% Income to Poverty Ratio	126.6 (125.0)	130.0 (127.2)	129.4 (215.3)
Education			
<8th grade	35.8	40.9	32.1
9-12th grade	48.0	44.5	52.9
College and above	16.2	14.5	15.0
Physical activity[1]	31.6 (4.9)	31.3 (4.8)	31.6 (4.5)
Cognitive Test Scores			
Verbal memory (16 pts)	8.1 (2.4)	7.8 (2.2)	8.0 (2.4)
Recognition (16 pts)	13.0 (2.7)	12.7 (2.6)	13.0 (2.7)
Executive functioning (#)	24.1 (10.4)	22.8 (10.2)	24.1 (10.3)
Mental processing speed (#)	22.9 (11.0)	22.8 (10.5)	23.8 (10.3)
Visuospatial (12 pts)	5.6 (3.1)	5.6 (3.2)	6.1 (3.1)

eTable 8. Summary of Participant Characteristics and Pollutant Concentrations by Tertile of 1 yr Average of Sulfur (ng/m^3)

**Study Characteristics**	**Lowest (<1008)** % or mean (SD)	**Middle (1008-1113)** % or mean (SD)	**Highest (>1113)** % or mean (SD)
Demographics			

Sex (% female)	69.3	71.4	70.7
Age (years)	55.6 (7.8)	57.9 (7.4)	57.5 (7.3)
% Income to Poverty Ratio	131.5 (240.0)	123.0 (120.3)	131.1 (127.4)
Education			
<8th grade	31.6	39.9	36.0
9-12th grade	52.5	45.1	49.1
College and above	15.9	14.9	14.8
Physical activity[1]	31.6 (4.9)	31.3 (4.3)	31.7 (4.9)
Cognitive Test Scores			
Verbal memory (16 pts)	8.1 (2.5)	8.2 (2.3)	7.8 (2.3)
Recognition (16 pts)	13.0 (2.7)	13.1 (2.7)	12.8 (2.7)
Executive functioning (#)	24.1 (10.3)	23.6 (10.7)	23.5 (10.1)
Mental processing speed (#)	24.6 (10.7)	23.0 (10.3)	22.5 (10.6)
Visuospatial (12 pts)	6.0 (3.1)	5.6 (3.1)	5.8 (3.2)
